# Understanding atypical eating behaviours in women with obsessive-compulsive disorder: A qualitative study

**DOI:** 10.1371/journal.pone.0328500

**Published:** 2026-06-10

**Authors:** Sonay Kucukterzi-Ali, Amanda K. Ludlow, Roberto Gutierrez, Naomi A. Fineberg, Tim M. Gale

**Affiliations:** 1 Department of Psychology, Sport and Geography, University of Hertfordshire, College Lane, Hatfield, Hertfordshire, United Kingdom; 2 School of Psychology, University of Sussex, Sussex, United Kingdom; 3 Hertfordshire Partnership University Foundation NHS Trust, Hertfordshire, United Kingdom; 4 Department of Clinical, Pharmaceutical and Biological Science, University of Hertfordshire, Hertfordshire, United Kingdom; Taipei Veterans General Hospital, TAIWAN

## Abstract

Atypical eating behaviours in adults with obsessive-compulsive disorder (OCD) are often overlooked or misdiagnosed, possibly due to some eating difficulties being synonymous with their associated OCD. This study explored eating experiences from the perspective of eleven females with OCD, aged between 20 and 45 years. A series of semi-structured interviews were conducted and analysed using reflexive thematic analysis. Three central themes were generated from the data: the hidden burden; the importance of control; the detrimental impact of OCD on eating. These themes encapsulated how prevalent atypical eating difficulties were among the females with OCD, often emerging in response to heightened states of emotions, the need for control and/or as a reflection of their OCD symptoms. Whilst the reported eating behaviours did not always meet the diagnostic threshold for an eating disorder, participants described them as posing significant challenges to their daily functioning, as well as negatively affecting those around them. The current findings suggest that individuals with OCD often perceive their eating behaviours to be directly associated with their OC symptoms, highlighting the need to acknowledge these challenges in clinical practice. A better understanding of the broader implications of OCD, particularly on eating behaviours, is warranted to facilitate tailored interventions and prevent additional complications associated with eating behaviours in this group.

## Introduction

Obsessive-compulsive Disorder (OCD) is characterised by the recurrent, persistent presence of obsessions (unwanted thoughts, urges or images) and/or compulsions, defined as repetitive behaviours or mental acts that are carried out to suppress such obsessions [1,2]. While there exists heterogeneity in the presentation of OCD symptoms, with obsessions varying across contamination, doubts and ordering, their frequency, intrusiveness and negativity are recognised as having a detrimental impact on an individual’s overall functioning and quality of life [3,4]. Importantly, in the context of the current study, its impact is thought to extend to patterns of behaviour in everyday activities, such as eating.

Obsessive-compulsive disorder and obsessive-compulsive (OC) symptoms have to date been associated with a range of atypical eating behaviours, which may vary in levels of severity. For example, individuals in the general population, who demonstrate elevated levels of OC symptomatology, are more likely to report increased atypical eating behaviours, such as selective eating [5–7], colloquially referred to as food fussiness, and pathological dieting [8]. One particular study also noted that selective eating during childhood was predictive of OC symptoms in later life, suggesting that early eating behaviours may be a marker for emerging OC symptoms [7]. Moreover, binge-purge behaviours [9] and intrusive thoughts about eating are also reported by those at risk of OCD to a greater extent than what has been seen in the general population [10]. Although such eating behaviours are deemed less severe than those observed in eating disorders, they can still have significant psychological and physical health implications, as well as substantially reducing quality of life [11–14].

Extreme clinical levels of eating disturbances are also noted to occur among some individuals with OCD. For example, co-occurring OCD and eating disorders are frequently observed in research and across clinical practice [15,16], with evidence suggesting that individuals with both conditions are more likely to experience increased levels of psychopathology (e.g., worse OC symptoms, anxiety, depression and post-traumatic stress disorder) compared to their counterparts with only OCD [17]. OCD and OC symptoms are associated with all types of eating disorders, including disturbances related to dieting (e.g., anorexia nervosa, bulimia nervosa and eating disorders not otherwise specified; [16,18–21]), bingeing (e.g., binge-eating disorder; [22]) and food avoidance (e.g., avoidant-restrictive food intake disorder; [14,23]). Moreover, it is proposed that those with an eating disorder are 8.9 times more likely to have OCD compared to non-clinical controls [24].

An additional complication in our understanding of the relationship between eating disorders and OCD is their overlap in symptomatology. For example, not only has OCD been observed across all eating disorders, but reductions in the symptomatology of one condition often correspond with reductions in the symptoms of the other, suggesting that eating disorder symptoms and OC symptoms are bi-directional [16,25]. However, there remains a critical gap in understanding atypical eating behaviours that are not within the remit of diagnostic eating disorder frameworks, such as selective eating, which has been recognised as having a detrimental impact on anxiety, mood and physical health.

Moreover, although research has demonstrated a link between OCD, OC symptoms and atypical eating behaviours, these studies have depended on the use of pre-existing questionnaires, such as the Eating Attitudes Test – 26 [26] or variants of the Eating Disorder Examination [27,28], which are not validated for use in OCD. Subsequently, the measures used may not comprehensively capture the atypical eating behaviours present in OCD or related to OC symptoms, highlighting the need for alternative methods to understand atypical eating patterns within this group. By taking a top-down approach—focusing first on eating disorders and then considering which groups are affected—there is a risk of missing or misinterpreting symptoms in individuals with OCD, whose eating concerns may present differently and not fit existing definitions of eating disorders. In contrast, addressing the issue from an OCD perspective allows for a better understanding of how eating concerns manifest in this population, as well as the unique pressures and barriers to seeking support that they may face.

To further our understanding of atypical eating behaviours and OCD, the current study aimed to qualitatively explore eating experiences in adults with OCD, including eating behaviours which may not meet diagnostic thresholds for an eating disorder. The study involved participants completing a one-off semi-structured interview exploring eating experiences in OCD and the impact of OCD on eating behaviours.

## Method

### Ethical approval

Ethical approval for this research was obtained from the University of Hertfordshire; Protocol Number: aLMS/PGR/UH/05462(3). The research was performed in accordance with the Declaration of Helsinki. All participants provided informed consent to participate in the study.

### Participants and sampling

Eleven participants, all female and aged between 20 and 45 years (M = 31.63, SD = 9.38), took part in the current study. Of the 11 participants, nine were from white ethnic and ancestral backgrounds (British n = 8; Irish n = 1) and one was from an Indian ethnic and ancestral background; one participant did not disclose this information. All participants, except one, had a formal diagnosis of OCD; the participant without a formal diagnosis had received treatment for OCD and was told by a healthcare professional that she had OCD. The decision to include participants without a formal diagnosis of OCD was to ensure the study was representative of the wider OCD population, where not all individuals with OCD are formally diagnosed, but have received treatment, or have been told that they have OCD by a healthcare professional.

Adults were recruited through purposive sampling via the charities OCD Action and the Orchard OCD Registry, as well as the social media platforms of X (formerly known as Twitter) and Instagram. The sample size was based on previous interview schedules with adults with OCD [29] and the ‘information power’ approach [30], that suggests the more relevant and information-rich your participants are, the fewer you may need. Here, the use of smaller samples can be justified when participants are highly relevant, provide rich, detailed data, and the study aim is narrow or focused (e.g., specific samples such as individuals with OCD and eating concerns). In such cases, a smaller, information-rich sample can yield sufficient insights without the need for larger numbers.

The group of participants scored above the clinical threshold (*M* = 29.2; *SD* = 19.57) of the Obsessive-Compulsive Inventory-Revised (OCI-R; [31]). Given that OC symptoms wax and wane in presentation, not all participants exceeded the OCI-R threshold at the time of completion (*n* = 4). Moreover, all participants reported having received treatment for OCD at some point during their lifetime, six of whom reported currently undergoing treatment for OCD (pharmacological intervention *n* = 4; psychological therapies *n* = 1; combined pharmacological and psychological intervention *n* = 1). Eight of the 11 participants reported a co-occurring condition; these details are presented in Table 1.

**Table 1:** Participant details.

Participant	Age (years)	BMI	OCD symptoms	Co-occurring conditions	Eating disorder history
A	22	19.1	Contamination fears	Anxiety	No diagnosed eating disorder but significant food anxieties
B	30	19.0	Contamination fears and health anxieties	Anxiety and depression	Self-identified eating disorder symptoms
C	41	22.6	Obsessions	Social anxiety disorder and depression	Self-identified food addiction to sugary foods and drinks
D	29	26.2	Magical thinking, checking	None reported	None
E	27	21.5	Harm avoidance obsessions	None reported	None
F	20	23.1	Obsessions, reassurance seeking	None reported	None
G	20	13.7	Contamination fears, obsession about control	Emetophobia, autism spectrum disorder, ADHD, anxiety and depression	History of eating disorders and working diagnosis of ARFID
H	38	25.2	Harm avoidance, health anxiety obsessions, contamination fears	Depression and generalised anxiety disorder	None
J	31	18.1	Contamination fears	Emetophobia, depression	Borderline anorexia nervosa during adolescence
K	45	22.3	Obsessions, reassurance seeking	Depression	Self-identified eating disorder symptoms during adolescence
L	45	30.4	Contamination fears, orderliness	OCPD, hoarding disorder	Self-identified binge-eating disorder

### Procedure and materials

**Fig 1:**
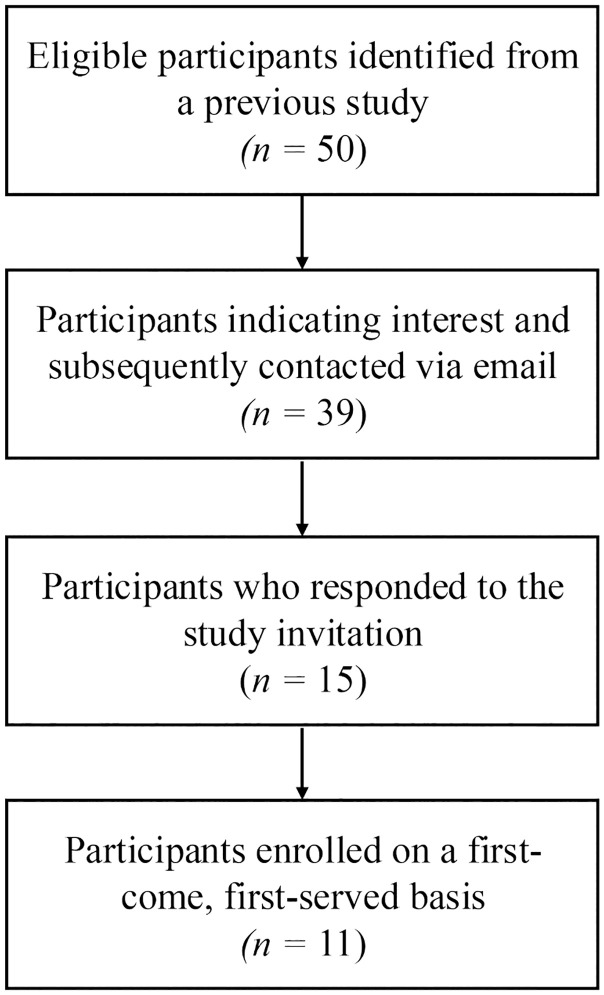
Participant selection process.

All participants had already participated in a previous quantitative study conducted by the research team, which involved the use of standardised measures to assess atypical eating behaviours. Participants from the previous study were asked to contact the lead researcher if they would be interested in taking part in a qualitative, semi-structured interview study. Those who expressed an interest were provided with written information about the current study via email. Fig 1 details the participant selection process. Participant consent was received using a verbal informed consent form and all interviews were conducted online; both of which were completed by the first author. Prior to beginning the interview, the researcher provided a brief introduction to the interview topic and the terms used (e.g., food fussiness), as familiarity with the research topic can enhance understanding of the interview response and coherence of the interpretation [32]. When participants were ready to proceed, they were informed that the digital audio recording and transcription would begin, and were reminded of their right to withdraw and confidentiality of their responses. Study recruitment and participation took place between 4^th^ January and 14^th^ May 2024.

A series of semi-structured interview questions and prompts were used during the interview. Findings from a prior study by the research team, which examined eating behaviours in adults with OC symptoms, as well as existing literature on eating behaviours in other co-occurring conditions (e.g., autism spectrum disorder), were used to inform the development of the semi-structured interview guide. Specific themes identified in earlier studies included the negative impact of clinical psychopathologies on eating behaviours and risk factors which may underpin atypical eating behaviours in clinical populations (e.g., specific OC symptoms, sensory sensitivities and perfectionism; [33–37]). These themes were explored in the current semi-structured interview, alongside core questions relating to diet and food preferences, childhood eating experiences, challenges with eating, the specific impact of OCD on eating and experiences with healthcare services.

The interviews ranged between 26 and 69 minutes. Upon completion of the interview, participants were thanked for their time and provided with a copy of their consent form. The University of Hertfordshire provided funding for the study; this was used to thank participants for their time with a £10 gift voucher. The funder was not involved in any aspect of the study design, delivery or publication.

### Data analysis

Qualitative reflexive thematic analysis was used to analyse the transcripts; this analytic approach considers existing knowledge on the topic area alongside the experiences outlined by participants [38]. Interviews were transcribed verbatim by the first author (SKA) and then read several times to enable familiarisation with the data prior to coding. Thereafter, a line-by-line process was used to generate codes, which were systematically documented. These codes were then collated into potential themes and subthemes, with clear documentation of how codes and themes were combined, refined or discarded throughout the analytic process. For example, several codes captured the compensatory nature of eating behaviours in OCD, as well as ways in which such behaviours could become maladaptive. These codes were interpreted as related to a broader concept of ‘control’, which subsequently informed the development of one of the themes.

The analysis process was discussed with the second author (AL) throughout to ensure transparency and that the coding and themes accurately represented the data [39,40].

Once the analysis was complete, participants were provided with a copy of the final results for member checking [41]. Member checking of the final themes allowed participants to confirm whether the analysis accurately reflected their experiences [42]. No changes were requested and participants agreed that the analysis provided an accurate summary of their experiences.

## Results

Three overarching themes were identified from the analysis of the interview transcripts; these are presented alongside eight sub-themes in Table 2. The first theme, ‘the hidden burden’, referred to the unrecognised eating difficulties faced by participants. ‘The importance of control’ highlighted the fine line between gaining and losing control when eating and, lastly, ‘the detrimental impact of OCD on eating’ described the negative effect of OCD on eating in day-to-day life. Each of these themes and subthemes are described below.

**Table 2:** Themes and subthemes of the analysis.

Themes and subthemes
The hidden burden
*1. Eating behaviours as a by-product of OCD symptomatology*
*2. It’s not always as it seems*
3. *Lack of support for eating*
The importance of control
*1. Compensatory control*
2. *Maladaptive attempts at stability*
The detrimental impact of OCD on eating
*1. Effects on daily living*
*2. Eating outside of home*
3. *Life through the lens of OCD*

### The hidden burden

The first theme represented the hidden eating difficulties faced by the adults with OCD. Many believed their OCD was a catalyst for their eating difficulties, but felt that these challenges were often overlooked in the support and/or interventions provided by healthcare services. Participants also spoke of a disconnect whereby visible manifestations of eating (e.g., eating healthily or being of a healthy weight) masked the challenges that they faced.

#### Eating behaviours as a by-product of OCD.

Although eating behaviours were not always the primary presenting issue, many adults reported that their eating was directly affected by their OC symptoms.

*In terms of the contamination, it has influenced what I eat… I’ve cut out things at times due to whatever was in the news (H)*

*If I thought I had any red on my plate, I would associate that as being something to do with blood and that it was contaminated, and I couldn’t eat it. (B)*

*[The OCD] routines transfer into other parts of life. So, if I’m preparing food, I have to do it in a certain way. (G)*

Moreover, participant E felt that she experienced more atypical eating behaviours when her OC symptoms were more severe: “*I would say definitely when my OCD is in general, more stressful, I have a lot more symptoms and then some of those symptoms are like the eating ones”.*

In cases of low mood or milder distress, participants would report more overeating as a form of comfort. However, when participants felt intense anxiety or distress, they were more likely to undereat.

*I think if I’m really distressed, it’s like you forget food. But if I’m like, just kind of low, I hit [sugary foods and drinks] like an alcoholic hitting the bottle (C)*

Similarly, participant D distinguished between when she would use eating as a form of comfort, and when she would be less likely to want to eat due to feeling distressed:

*If I’m stressed and I don’t know what to do with myself, or if I’m feeling guilty or particularly worried about something and a bit restless, then I probably want to eat. But, if I’m tired and frustrated, and maybe more tearful, then maybe I’d be less inclined to eat (D)*

#### It’s not always as it seems.

Another commonality among participants was that their difficulties with eating were not always visible. Subsequently, this may have prevented others, or even themselves, from identifying issues with their eating behaviours or diet. For example, participant H would receive compliments on her appearance, despite her weight loss being driven by her OC symptoms: *“lots of people would comment on it and they think oh, you look great, but I’m actually really unwell”.*

Similarly, participant C described her appearance as fit and healthy, despite acknowledging that her diet was restricted and largely consisted of sugary foods and drinks: *“If someone looked at me, they would think I was fit and healthy”.*

Some participants mentioned that their perceived balanced or healthy diet may have masked their actual challenges with eating:

*I try to keep it balanced somehow … but at the same time it’s restricted because the only way it finds the balance is through me doing everything in the background to freeze everything or to buy everything that’s got really long dates (G)*

Another participant, B, suspected that she had symptoms of an eating disorder, but she perceived these to be less serious as her diet was healthy:

*Sometimes I think I have a borderline eating disorder issue. It’s not like out of control because I eat a healthy diet and I’m not unwell (B)*

#### Lack of support for eating.

Most participants felt that they did not receive support for their eating difficulties, despite expressing their challenges to healthcare professionals. For example, G, who had a history of an eating disorder, described how her thoughts about her eating behaviours were not considered:

*I only came to the conclusion that [my eating] was linked to OCD myself. I had to figure everything out myself. I had to put all the puzzle pieces together myself. It was really confusing and distressing to do so. But even when I did, they still didn’t listen to me, and they still didn’t even offer me different care or consideration when treating me (G)*

Another participant also described concerns about her weight, but reported a lack of support from healthcare professionals:

*When I started with my psychiatrist, I was very self-conscious of losing a lot of weight and it would have been something I would have probably brought up more than being asked, but I didn’t get any support around it (H)*

Participant K, who suspected that she had an eating disorder during adolescence, also felt that her difficulties with eating were unsupported by healthcare professionals. She suggested a necessity for healthcare professionals to understand how OCD affects individuals beyond their primary OC symptoms and to understand how the patterns of OCD may be linked to pathological eating patterns:

*What I’d really want them to think about is not just what’s presented in front of them in that moment, but to consider how some of these other things and the thinking patterns and the mental processes behind them could actually be quite similar (K)*

Participant L, who self-identified as having binge-eating disorder, had previously sought support for her eating behaviours, but was told that she had already received a similar treatment for OCD. She also reported being told that the eating disorders service would not be able to support her as her BMI was in the high category:

*I did ask them specifically about the eating disorders pathway and if that’s something I could access for binge-eating disorder. They said the treatment would be [cognitive behavioural therapy] and you’ve had [cognitive behavioural therapy]… I did specifically inquire about eating disorder services, but I was told that the eating disorders services were only for people with low BMI and not those with high BMI (L)*

In the few cases where support was provided, participants felt disappointed that it did not consider the effect that OCD has on eating. For example, J, who is of low weight and has a limited diet due to fears of contamination and emetophobia, was referred to a dietician who suggested that adding more calorie dense products to her food would help her gain weight. However, J would have preferred support which helped her to navigate difficulties with food preparation and eating: *“I was referred to a dietician actually and I didn’t find it helpful at all”.*

*I’ve had all sorts of [diabetes] education, but I have a problem with preparing [food]. I know it’s unhealthy to eat processed foods and ready meals, but I haven’t managed to overcome food preparation (L)*

### The importance of control

The need for control when eating or preparing meals was a recurring pattern identified among the participants. This included eating certain foods or having certain rituals around eating. However, in some cases, participants identified that their attempts to gain control became overwhelming and restrictive, indicating a fine line between gaining control and losing control.

#### Compensatory control.

Participants’ eating behaviours were often adopted as a method of creating a sense of safety, stability, and connection in the present moment, especially when things felt overwhelming, chaotic, or uncertain. Some participants, such as participant A, described that she would resort to safe food during times of distress: *“If I’m very stressed or if I’m feeling very anxious, I do have certain foods that I’ll go back to”.*

For some, specific routines around food provided them with a sense of consistency during uncertain times. For example, participant F reported wanting to eat the same foods when her grandmother was in hospital: *“every single day after that I really wanted that one meal every single day just to form a bit of a routine”.*

Some participants also tried to control and prevent the negative consequences of eating, which included vomiting or becoming unwell. For example, A would eat small bites of food to avoid choking: *“I’d take really small bites, like microscopic. That’s why it would take me so long to eat. I just was so scared of choking”.*

Compensatory behaviours, such as restrictive dieting commonly observed in eating disorders, were also noted by a few participants. For example, B described feelings of disgust after eating something she perceived to be fattening and had the urge to control this by restricting later meals.

*I just kept feeling really disgusted that I just sat and ate that. I would also feel really fat. I’d probably be thinking of the next meal and how I’m going to compensate for having eaten that (B)*

In contrast, E explained that her eating behaviours were not used to compensate for weight gain. Rather, she described that eating behaviours were something she could gain control over to provide a sense of security:

*I’ve teetered on the edge of having eating symptoms and stuff, it’s not because I think I’m going to get fat or anything. It’s because it’s just something that I can very easily control (E)*

#### Maladaptive attempts at stability.

Whilst some participants used their eating behaviours as a method of providing some certainty, some acknowledged that the restrictions posed by their OC symptoms led them to lose control of their eating behaviours. Participant J explained that her OCD rituals concerning food preparation were initially used to gain control over becoming unwell, but recognised that these rituals were controlling her instead: “*You take all these steps to make sure you do have control, but actually, if you think about it, the OCD is controlling me”.*

She further described closely monitoring the pace of eating, due to fears of becoming unwell, illustrating how attempts to maintain control could become restrictive:

*I have to watch the clock to see what time it was when I started eating to when I finish. I’ll go slower if I feel like I’m eating too quickly, because the worry is if I eat too quickly, I’ll be sick. (J)*

Similarly, G described that she was unable to deviate from set routines involving food and meal preparation, indicating that she could not control her thoughts and compulsions:

*I’m not just restricted by the type of food I eat or how I eat and things like that, I’m also restricted by time. So physically my body, my anxiety will not let me eat after 7:00 o’clock in the evening. Because for no reason whatsoever, 7:00 PM, even a second past, even 7:00 PM on the dot, I can’t eat past 7:00 PM. (G)*

Participant K noted that her desire to have a consistent eating routine was driven by anxiety. She did not want to miss her meals as this could lead to feeling a lack of control, which she found stressful:

*I can get quite anxious if I miss a meal as well. I find that really stressful. I don’t quite know what I think’s going to happen. (K)*

Participant C also described that her eating behaviours were not controlled: “*I’ve got an addiction and it’s hard to stop it”.*

### The detrimental impact of OCD on eating

The final theme represented the difficulties that OCD posed on eating, including the negative impact on daily life, as well as eating out of the home.

#### Effects on daily living.

Many of the participants reported that the impact of OCD on their eating also came with negative effects on their daily lives. For K, her obsessive symptoms led her to have increased structure around her daily eating routines:

*I can remember my day becoming structured around when I was going to be able to have something to eat and what that was going to be, and how I was going to avoid a situation where I might have to veer from that structure. (K)*

Some participants also noted that not only did their OCD have a negative impact on food preparation and dietary intake, but also recreational activities concerning food. For example, B stopped baking: “*I never thought that I would be able to bake again because of the thoughts I had to do with contamination of food”*. Participant J engaged in reassurance seeking behaviours during food preparation as she questioned whether she would become unwell from undercooked food:

*I do seek a lot of reassurance as well when I’m cooking or when I’m eating food. I will ask, does this look right? Does this taste right? Are you sure this is cooked properly? How long did you cook it for? And then I’ll ask again. Will this make me ill? (J)*

Similarly, participant L’s cleaning obsessions made it difficult for her to use the kitchen, which meant that she was unable to cook healthy and nutritious meals. Consequently, she often resorted to processed foods which she acknowledged were not ideal for her health:

*I don’t actually like [ready meals and processed foods], it’s just the convenience of having it and the difficulty I have with making other types of food. If I prepare my own food, I have to sanitize the work surfaces, I’ll have to clean the utensils and clean the kitchen and put them away afterwards. It’s so exhausting, and then you have to deal with the leftovers (L)*

Some participants also felt guilty that their difficulties with OCD and eating had a negative impact on those around them:

*It doesn’t just affect me, but also the people around me. That brings on a lot of guilt and, in turn, just makes the routines more restrictive because I want to protect the people around me. But, it’s restricting them. (G)*

*It’s not just impacting me, but others around me, which is really sad. My husband gets really anxious cooking because he doesn’t know if I’m going to eat it or not, so now he carries out safety behaviours by overcooking food. (J)*

#### Eating outside of the home.

Eating outside of the home was further complicated by OCD, with many participants expressing concern around feeling unsafe outside of the controlled home environment. For example, participant A, who experienced eating difficulties from a young age, felt that she was unable to challenge her obsessions when eating outside of home: *“At home obviously it’s a very safe environment for me to challenge myself in, whereas outside it’s not always”.*

Similarly, participants described feeling anxious when eating out and would often try to find ways to make the situation safe out of fear of feeling unwell:

*Even if I was going out with my friends or something, I wouldn’t have been able to. Even if we were going out for pizza, it would have been really stressful. I would have been thinking about what I could do to try and make it safe for me (K)*

*Eating out somewhere new fills me with anxiety, because if someone’s not recommended it, it’s that thought of ‘what if I get food poisoning?’ So, before I go out to a restaurant, I will look at their hygiene rating and then if it’s a four or five, I’ll be like okay, that’s fine. (J)*

For G, eating outside of the home caused much distress, leading her to engage in checking behaviours to ensure her environment was clean. Although she recognised that this was not socially appropriate, she would often be unaware of doing this as it became habitual:

*I’m on really high alert and I check everything around me… even unconsciously, if I walk into someone else’s kitchen, I will impulsively just check around the kitchen to make sure everything’s clean…I know that it probably seems really rude and invasive, but I can’t help it and a lot of the time I don’t notice I’m doing it because I’m so used to doing it at home for myself. (G)*

In contrast, some participants enjoyed eating outside of the home as it provided a distraction from their OCD thoughts. Participant L, who had difficulties with preparing food, liked to eat out as she would not need to worry about cleaning up: “*If you eat out you don’t have to worry about washing up”.*

*I find it distracts me from the OCD and that the OCD voice is a lot quieter because there’s other voices [socialising noise] in the room. I wouldn’t avoid socialising for food or anything like that, I’d quite enjoy going to a restaurant (D)*

#### Life through the lens of OCD.

Some participants offered an explanation as to why OCD impacted their daily lives and eating behaviours. They described that their perceptions of the world were altered by their OCD, which led them to doubt reality and question things that someone without OCD would not. For example, L recognised that her difficulties using the kitchen were constrained by rigid rules which she had to follow, whereas this may not be an issue for someone without OCD:

*A person without OCD would be like okay, this is what they recommend for food hygiene purposes, and this is what I can manage – they can take the advice within reason, but it just gets a bit blurred with my OCD (L)*

Participant G also felt that her perceptions of reality were skewed by OCD. Although she recognised that her thoughts may be irrational, she could not escape the doubts:

*Realistically, I know it’s a professional chef in a clean kitchen. Otherwise, the restaurant would be shut down, but in the little OCD corner of my brain, it just finds so many excuses to tell me that I’m in danger or that it’s going to make me unwell, or it will make me anxious so that I feel nauseous and stop eating. (G)*

Similarly, J described her dread of mealtimes as she was never sure what compulsion she would have to carry out to feel safer around food*, “Every mealtime you dread, because I can’t say what my brain is going to start telling me what I need to do.”*

Whilst F reported very few atypical eating behaviours herself, she provided insight as to why those with OCD might have difficulties around food*, “If [those with OCD] struggle with eating, it’s probably due to a compulsion or intrusive thoughts.”*

## Discussion

This study aimed to qualitatively explore atypical eating behaviours among individuals with OCD. All participants in the study described some degree of atypical eating behaviours which appeared to align with specific symptoms of OCD; for example, engaging in food avoidance and food preparation rituals to avoid becoming contaminated or feeling unwell. Yet, for many of these individuals, their primary OC symptoms and their ability to maintain a healthy bodyweight and/or diet often masked their eating difficulties, despite having severe implications for their everyday functioning and psychological wellbeing.

In the current study, many attempted to gain control over their obsessions or uncertainty in the environment by engaging in food-related rituals or compulsive eating behaviours. Previous research also suggests that control-related beliefs are considered a maintenance factor of OCD [43,44]. It has been proposed that seeking control serves to avoid the distress caused by obsessions, internal emotions and the uncertainty of one’s environment. In addition to OCD-specific mechanisms, such eating behaviours may also be understood in the context of transdiagnostic frameworks. For example, difficulties with emotion regulation may underlie the use of rigid or ritualised eating behaviours as means of managing distress or uncertainty [45,46]. Similarly, perfectionistic tendencies, which are commonly observed across both OCD and eating disorders, may contribute to rigid rules around food, eating pace or preparation [33,35]. For example, participant B would engage in compensatory behaviours to control her anxiety around becoming ‘fat’, and participants G and J would carry out food preparation rituals to avoid the anxiety of becoming unwell.

Interestingly, whilst gaining control appeared to be a catalyst for atypical eating, some participants acknowledged that they felt trapped within their rigid routines around eating, suggesting that their eating behaviours were ultimately controlling them. These findings echo existing research which proposes that, although individuals with OCD have a desire to control their thoughts, they have a lower sense of control over their thoughts (i.e., obsessions) or their ability to cope with particular situations, which motivates the use of short-term strategies (i.e., compulsive behaviours) to regain control [47]. From a transdiagnostic perspective, this pattern may also reflect maladaptive emotion regulation strategies, whereby control-based compulsions or ritualistic behaviours temporarily alleviate distress, but reinforce longer-term challenges. Subsequently, engagement in compulsive and/or ritualised eating behaviours may provide an illusory sense of control over obsessions; however, what began as a strategy for control might lead to losing autonomy over eating behaviours, indicating the presence of a vicious cycle.

Participants also spoke of how their ritualised eating behaviours and food preparation routines caused difficulties for not only themselves, but those around them. Such behaviours also posed challenges when eating outside of the home, which would often lead to engagement in safety behaviours or avoidance. These findings extend previous research which highlights that OCD is associated with significantly impaired functioning in everyday life, including the ability to uphold employment and maintain relationships [48–51].

Whilst many participants perceived their OCD and atypical eating behaviours to have a detrimental impact on their day-to-day functioning, they felt their eating issues were not supported by healthcare professionals. Even in severe cases of atypical eating, where support was provided, participants felt that interventions were not tailored to support their eating difficulties which were complicated by OCD. For example, in accordance with National Institute for Health and Care Excellence guidelines [52], participant L was provided with educational resources for her diabetes diagnosis. However, L’s difficulties with eating a healthy diet were not related to her lack of knowledge around eating well; instead, her difficulties stemmed from concerns over contamination and routines around food preparation which led her to consume convenient, processed meals. Therefore, it would be important to incorporate the perspectives and unique needs of those affected in treatment decisions to improve intervention outcomes, and ensure that the principles of patient-centred care are upheld [53–55]. Moreover, given the consistent reporting of atypical eating behaviours and their impact on daily functioning, there is a need to evaluate whether individuals with OCD should be routinely screened for such behaviours in clinical practice. Introducing these measures may help to identify pathological eating patterns earlier, which could prevent associated health risks, as well as the development of eating disorders and more complex psychopathologies [13,56–58].

The current findings should be interpreted within the context of some limitations. For example, qualitative research often requires a homogenous participant sample, however the participants demonstrated heterogenous presentations of OCD, such as contamination concerns, obsessions and magical thinking. Given that earlier research has linked specific OC symptoms (e.g., cleanliness) to atypical eating behaviours, it would be of interest to examine eating experiences within specific presentations of OCD; such explorations may help to shed light on whether individuals with certain OC symptoms are at greater risk of atypical eating [33,36,59].

Additionally, there are shortcomings in our methods of recruitment to note. For example, some participants were recruited after completing a quantitative survey on eating behaviours. This may have resulted in participant-led biases, including participants altering their behaviour at interview to be consistent with how they responded in the quantitative surveys, rather than reflecting their true, current feelings. However, researcher-led biases were minimised through not analysing the survey data until after the qualitative data and analysis had been completed. There was also likely to be some inherent survey bias, with participants drawn from the survey inheriting the same sampling biases (i.e., non-representative) as the initial survey. This may have resulted in an overrepresentation of adults with more marked symptoms and eating-related difficulties than if sampling from only the general population [60]. Also, all participants were female and from predominantly white backgrounds, which limits the understanding of eating experiences in the broader OCD population. It is particularly important for future research to address atypical eating in more ethnically diverse populations as there is evidence to suggest disordered eating may be more prevalent within these groups [61]. Similarly, greater attention is needed for males with OCD, who are more likely to develop an eating disorder, compared to their female counterparts, and are often reported to be overlooked for eating disorder support [62–64].

In summary, this was the first study to provide unique insight into the eating experiences of females with OCD and the complex interplay between OC symptoms and atypical eating behaviours. Using a qualitative design, participants were able to describe their eating difficulties beyond the constraints of existing atypical eating measures. Whilst only a few of the participants had a history of eating disorders, many reported debilitating eating behaviours, such as food avoidance, ritualistic eating and loss of autonomy when eating or preparing foods. While many of these eating behaviours could be considered secondary to OCD, they may also be maintained by broader transdiagnostic processes that are commonly observed in eating disorders, such as difficulties with emotion regulation or perfectionism. Participants highlighted that their eating behaviours were closely intertwined with their OCD, despite atypical eating behaviours not being recognised as a core symptom. Consequently, challenges associated with eating were often overlooked in healthcare settings, highlighting a critical gap in clinical awareness and the need for more inclusive, targeted interventions. Future research should examine how OCD affects eating behaviours across more diverse populations and ensure that support reflects the complex challenges that may not fall within the boundaries of traditional diagnostic frameworks.
